# Ovarian tissue bank

**DOI:** 10.3325/cmj.2021.62.297

**Published:** 2021-06

**Authors:** Katarina Bilić, Marija Vilaj, Branka Golubić-Ćepulić, Davor Ježek

**Affiliations:** 1 Zagreb University School of Medicine, Zagreb, Croatia; 2 Department of Transfusion Medicine and Transplantation Biology, University Hospital Center Zagreb, Zagreb, Croatia; 3 Department of Histology and Embryology, Zagreb University School of Medicine, Zagreb, Croatia; 4 Scientific Center of Excellence for Reproductive and Regenerative Medicine, Zagreb University School of Medicine, Zagreb, Croatia *davor.jezek@mef.hr*

Medicine has constantly been trying to prolong the human lifespan and improve the quality of human lives. Significant advances in malignant diseases treatment have decreased cancer mortality rate. However, medications and procedures used to fight cancer may also leave unwanted side effects, especially when it comes to fertility. This is why preserving fertility in patients undergoing aggressive treatments for cancer, or even benign diseases, is becoming a new priority in reproductive medicine.

A promising procedure, ovarian tissue cryopreservation (OTC), has been offered to women in addition to commonly used oocyte cryopreservation (OOC) and embryo cryopreservation (EC), with the aim of preserving fertility when gonadotoxic treatment is indicated. Furthermore, OTC is the only fertility preservation option available for prepubertal patients since it does not require ovarian stimulation and oocyte harvesting. Instead, only unaffected ovarian tissue fragments are retrieved from the patient (ideally before the administration of gonadotoxic medications). Biopsy retrieval surgery is performed laparoscopically, not leading to severe surgical complications. In most cases, a unilateral or bilateral ovarian biopsy is performed. However, when the patient is a prepubertal girl, the gynecologist sometimes opts for unilateral oophorectomy to gather enough tissue. The collected ovarian tissue is then processed, carefully examined, cryopreserved, and stored for future use.

Tissue cryopreservation presents a crucial moment in the entire process. Nowadays, a slow freezing method is most frequently used since it has been thoroughly tested and has shown good results. The slow freezing protocols for human ovaries are based on a gradual temperature drop within predefined time intervals. On the other hand, vitrification is based on an ultrafast cooling rate combined with a high concentration of cryoprotectant agents (CPAs). The procedure is quick and demands less expensive equipment, but it is not risk-free since high CPAs concentrations can be toxic to the cells. Additionally, the cooling process, although fast, is not easy to perform, making vitrification still not widely used. Although a limited number of reports suggests its efficiency, there has been no standard vitrification protocol for ovarian tissue.

As the ovary tissue is being cryopreserved, a sample is sent for histopathological analysis to determine the primordial follicle count and exclude the presence of malignant cells. When the patient is cured of her malignant disease and desires to conceive, her hormonal status, risk of malignant relapse, and overall physical status is assessed in collaboration with oncologists. The cryopreserved ovarian tissue fragments are then thawed to be reimplanted in orthotopic or heterotopic sites in the patient’s body. After orthotopic autotransplantation, the tissue is placed into the remaining ovary, ovarian fossa, or broad ligament, a process requiring an invasive surgical approach under general anesthesia. However, these sites provide a favorable environment for follicular/oocyte development and a possibility for natural conception, as confirmed with more than 150 live births worldwide (1). Heterotropic transplantation is less invasive and frequently does not require general anesthesia since the frozen-thawed cortical tissue can be placed into the subcutaneous space of the forearm, subcutaneous tissue of the abdomen, the anterior wall of the abdomen, beneath the peritoneum, or even in the rectus muscle. However, these sites do not provide an environment as beneficial as that provided by orthotopic transplantation. Consequently, the quality of the produced oocytes could be reduced, natural conception is impossible, and patients require *in vitro* fertilization to conceive. Endocrine function in patients is often restored, but there are not as many reports of live births. Considering all this, the approach to the stored tissue reimplantation should be decided upon for every patient individually. Regardless of the chosen procedure, patients are usually grafted with 1/3 to 1/2 of their preserved ovarian tissue if the transplantation procedure has to be repeated (2). The life span of ovarian grafted tissue differs from patient to patient, with the usual duration of the graft activity being over four years if no chemotherapy was performed before tissue collection. Still, in some cases, the graft life-span was even close to 10 years (3).

**Figure 1:**
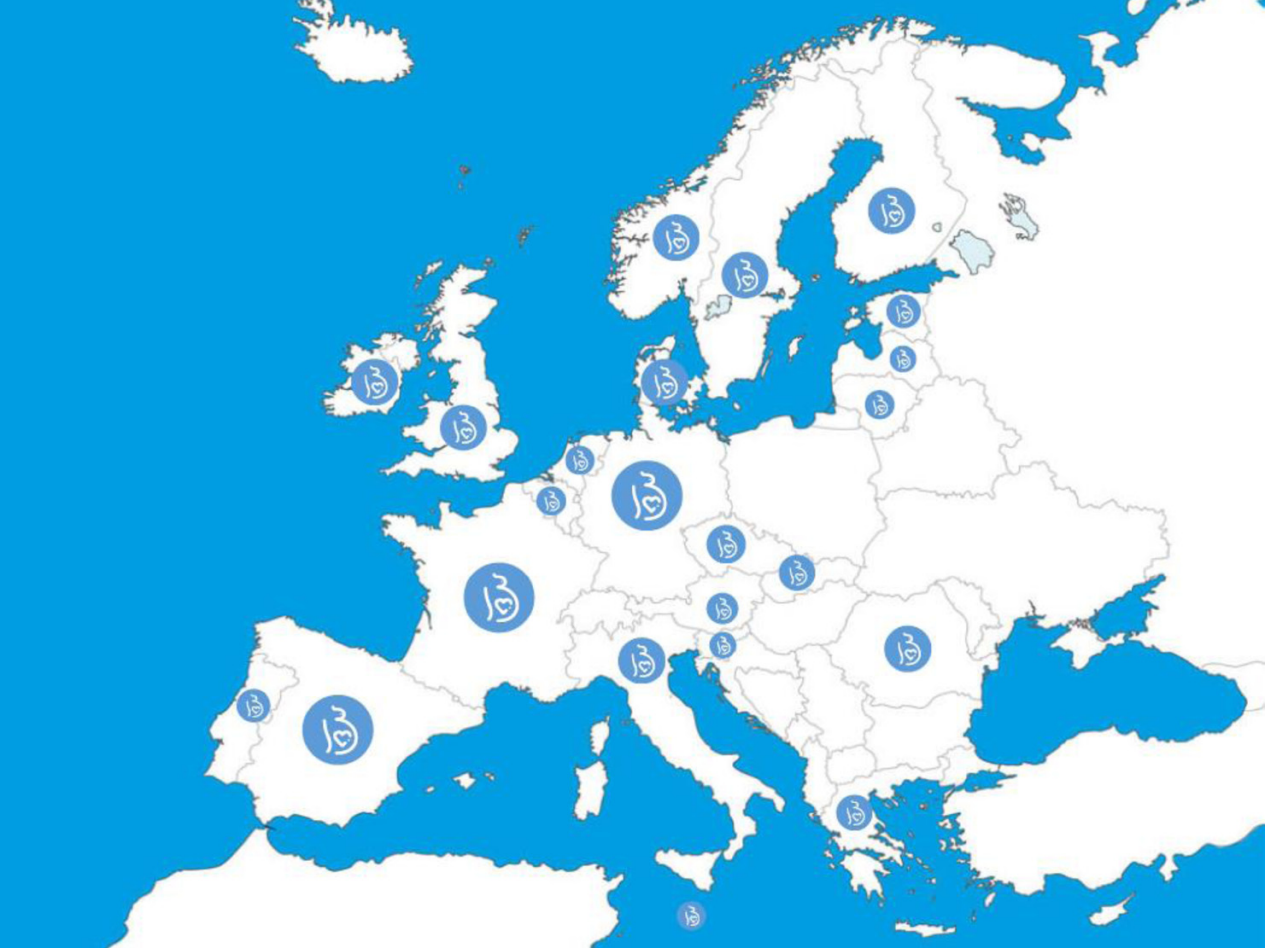
The distribution of ovarian tissue banks in the European Union and the United Kingdom.

Nowadays, many women choose to postpone childbirth due to social reasons. On the other hand, with increasing age, the chances of conception lessen since the quantity and quality of follicles decreases. Bearing this in mind, ovarian tissue (while it still holds excellent proliferation potential) can be preserved and reimplanted once social circumstances allow women to plan pregnancy and combine career and motherhood. Many European hospitals and fertility centers have included OTC in their fertility preservation programs alongside commonly used methods, such as OOC and EC. Today, ovary tissue banks already exist in 23 European countries (Figure 1), and Croatia is proud to become the 24th.

The first child conceived from frozen-thawed ovarian tissue was born in 2004 (4), and since then, OTC has resulted in more than 150 live births worldwide (5). Excellent results were presented in 2017 in a cohort of 545 patients who underwent OTC, with a pregnancy rate of 33% (6). Concerning the graft hormonal function, a renewed ovarian endocrine function was reported in 95% of the women (5). Interestingly, puberty initiation after the transplantation is also possible in patients who underwent OTC before starting their hormonal maturation. Therefore, autotransplantation of frozen-thawed ovarian tissue has helped to restore endocrine function both before and after puberty.

Considering all the studies and promising results, OTC as a fertility preservation and restoration technique is now ready to be translated into clinical practice and is no longer considered an experimental technique. Fortunately, the American Society for Reproductive Medicine has recently removed it from the list of experimental treatments (7).
